# Supplementary material for: Trends in non-daily cigarette smoking in England, 2006–2024
Source: BMC Med. 2024 Oct 24;22:426. doi: 10.1186/s12916-024-03635-1 (PMC11515449; doi:10.1186/s12916-024-03635-1)
Supplement: Supplementary file 1 — Additional file 1. Tables S1-S2, Figs. S1-S7. TabS1—Model selection: AIC values for models with 3, 4, and 5 knots. TabS2—Modelled estimates within age groups of the prevalence of non-daily smoking among adults in the first available monthly wave and in April 2024. FigS1—Prevalence of daily and non-daily smoking, by age. FigS2—Trends by age in non-daily smoking among adult cigarette smokers in England, stratified by vaping status. FigS3—Prevalence of daily and non-daily smoking, by gender. FigS4—Prevalence of daily and non-daily smoking, by occupational social grade. FigS5—Prevalence of daily and non-daily smoking, by vaping status. FigS6—Trends by vaping status in non-daily smoking among adult cigarette smokers in England, stratified by age. FigS7—Prevalence of daily and non-daily smoking, by level of alcohol consumption. [file 12916_2024_3635_MOESM1_ESM.pdf]

Table S1. Model selection: AIC values for models with 3, 4, and 5 knots

	AIC		
	3 knots	4 knots	5 knots
Adults			
Overall	84094.21	84096.22	84098.73
By age	81001.88	81005.57	81002.81
By gender	83563.32	83566.09	83568.33
By social grade	84081.01	84076.38	84053.82
By vaping status	79759.90	79760.93	79734.71
By alcohol consumption	47506.86	47505.12	47509.13
Cigarette smokers			
Overall	52417.53	52419.16	52421.58
By age	51445.00	51447.41	51450.59
By gender	52180.45	52184.20	52188.67
By social grade	51455.64	51434.93	51433.83
By vaping status	50833.76	50835.59	50829.92
By alcohol consumption	28024.34	28021.74	28019.87

AIC, Akaike Information Criterion.

Shaded cells indicate the best fitting model (the model with the lowest AIC or the simplest model within 2 AIC units).

Table S2. Modelled estimates within age groups of the prevalence of non-daily smoking among adults in the first available monthly wave and in April 2024

	Adults		Cigarette smokers	
	Prevalence, % [95%CI][1]		Prevalence, % [95%CI][1]	
	Nov 2006	Apr 2024	Nov 2006	Apr 2024
Age group (years)				
18–24	4.6 [4.1–5.1]	10.1 [9.3–11.1]	14.7 [13.3–16.4]	48.2 [45.3–51.1]
25–34	3.8 [3.5–4.1]	6.9 [6.5–7.3]	12.5 [11.6–13.5]	36.0 [34.2–37.8]
35–44	3.0 [2.7–3.2]	4.3 [4.0–4.7]	10.5 [9.6–11.4]	24.8 [23.2–26.5]
45–54	2.2 [2.0–2.5]	2.8 [2.5–3.0]	9.2 [8.3–10.1]	18.2 [16.7–19.7]
55–64	1.6 [1.4–1.8]	1.8 [1.7–2.0]	8.5 [7.7–9.5]	15.2 [13.9–16.5]
≥65	0.8 [0.6–1.0]	0.9 [0.8–1.2]	8.3 [6.3–10.9]	14.1 [11.3–17.5]

[1] Data are weighted estimates of prevalence in the first and last months in the study period from logistic regression with survey month and age modelled non-linearly using restricted cubic splines. They are calculated as means of modelled estimates across years of age in each age group (e.g., prevalence among 18-24-year-olds was calculated as the mean of modelled estimates for those aged 18, 19, 20, 21, 22, 23, and 24 years).

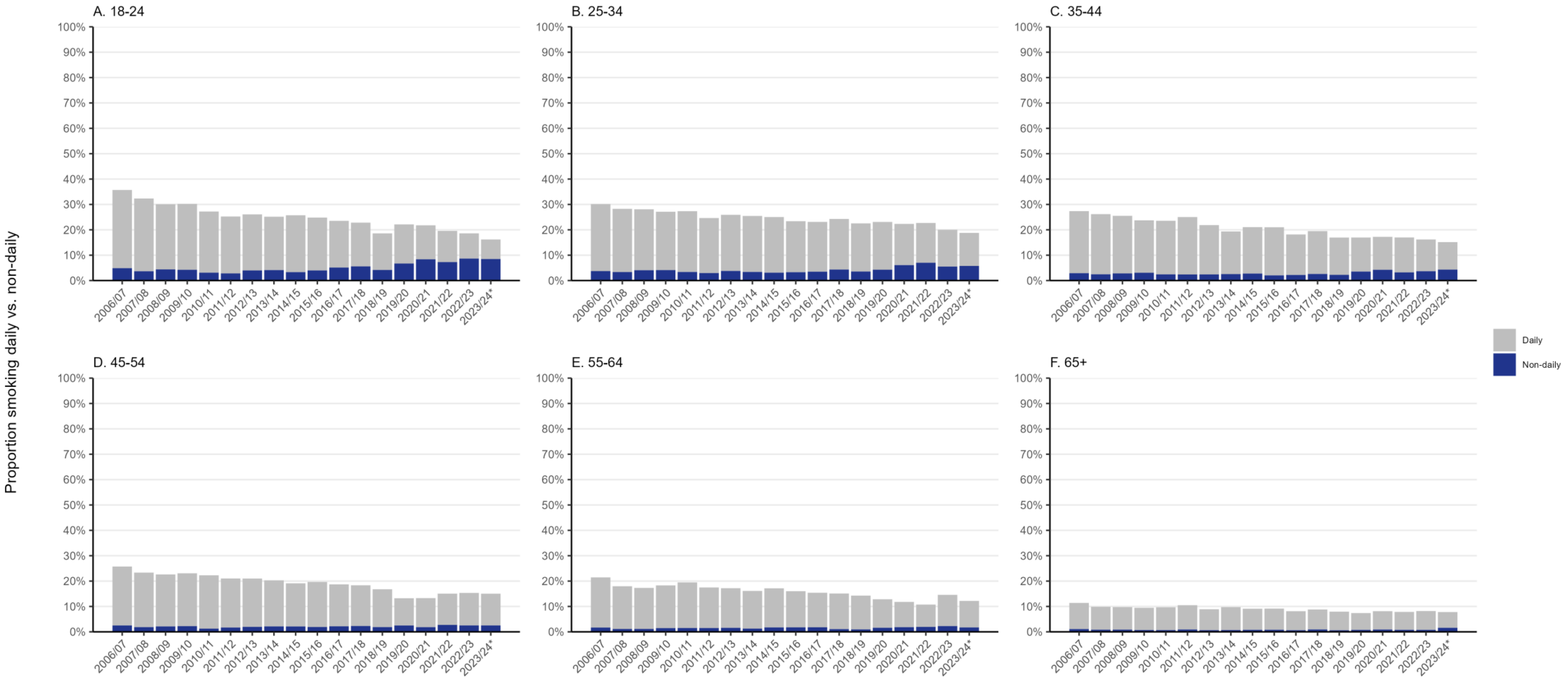

Figure S1. Prevalence of daily and non-daily smoking, by age.

Panels show weighted data aggregated by year among adults in England. Bars represent the proportions smoking daily and non-daily. Data are aggregated across 12-month periods (November to October). *Data for 2023/24 are based on November to April only.

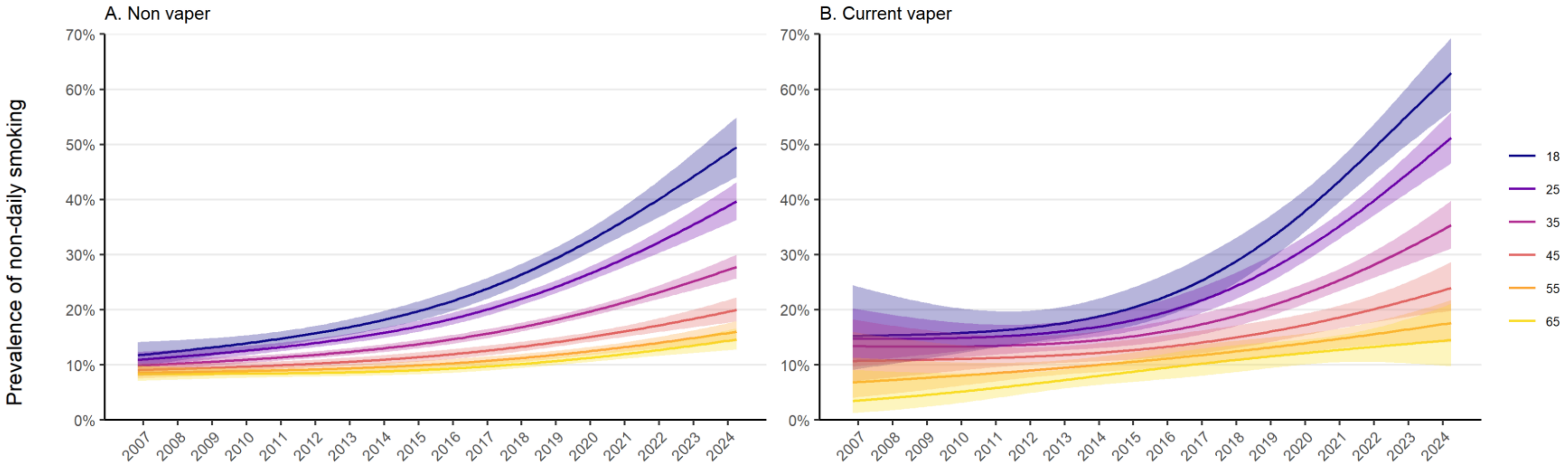

Figure S2. Trends by age in non-daily smoking among adult cigarette smokers in England, stratified by vaping status.

Lines represent the modelled weighted proportion by monthly survey wave (modelled non-linearly using restricted cubic splines; three knots) and age, stratified by vaping status. Shaded bands represent 95% confidence intervals. Note: data on vaping status were only available for cigarette smokers from April 2011.

Unweighted sample sizes: non-vapers *n*=34,566; current vapers *n*=7,693.

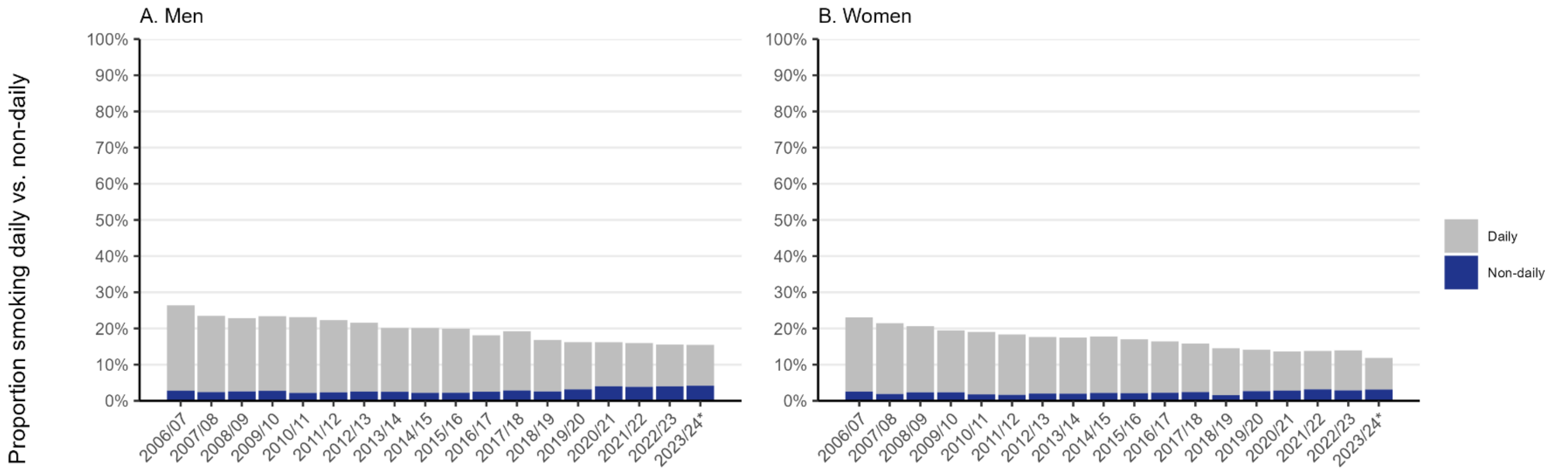

Figure S3. Prevalence of daily and non-daily smoking, by gender.

Panels show weighted data aggregated by year among adults in England. Bars represent the proportions smoking daily and non-daily. Data are aggregated across 12-month periods (November to October). *Data for 2023/24 are based on November to April only.

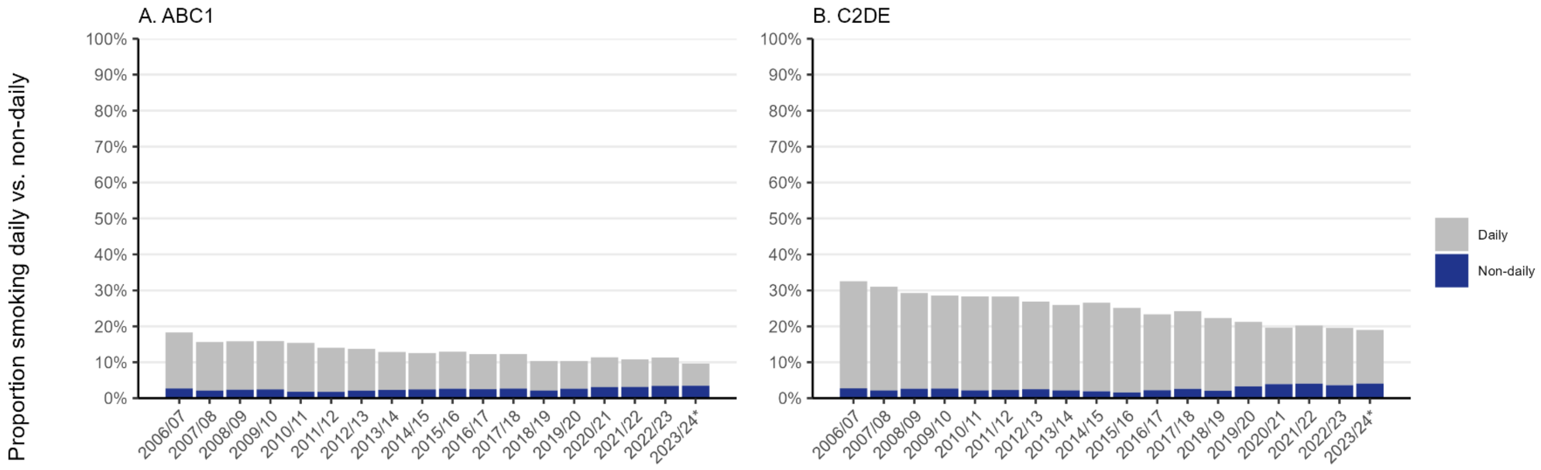

Figure S4. Prevalence of daily and non-daily smoking, by occupational social grade.

Panels show weighted data aggregated by year among adults in England. Bars represent the proportions smoking daily and non-daily. Data are aggregated across 12-month periods (November to October). *Data for 2023/24 are based on November to April only.

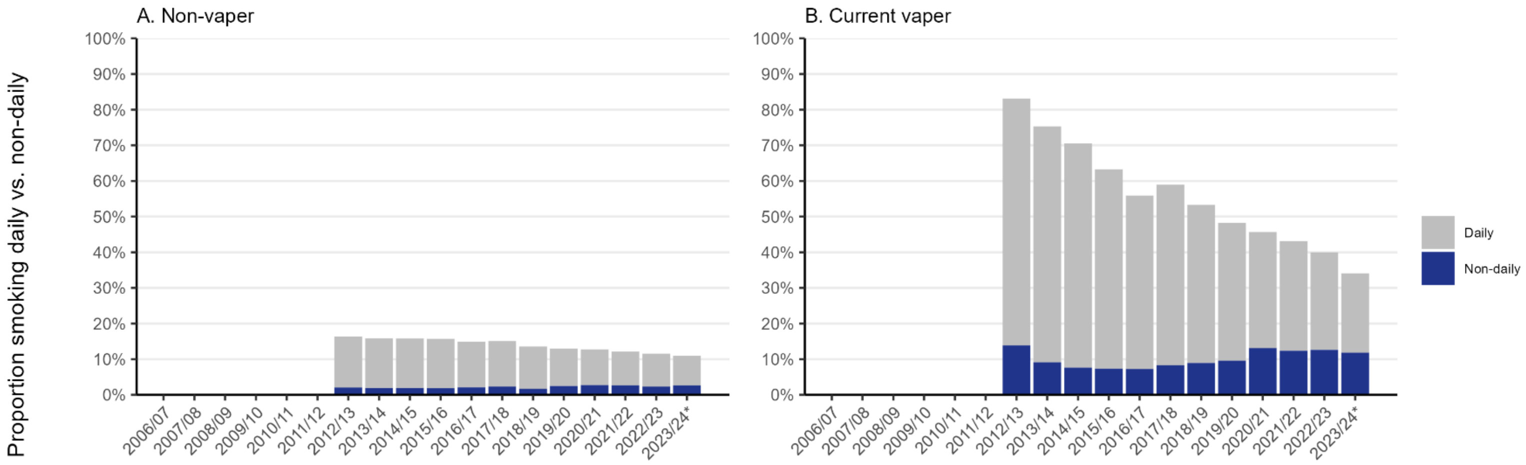

Figure S5. Prevalence of daily and non-daily smoking, by vaping status.

Panels show weighted data aggregated by year among adults in England. Bars represent the proportions smoking daily and non-daily. Data are aggregated across 12-month periods (November to October). Data on vaping status were collected from October 2013. *Data for 2023/24 are based on November to April only.

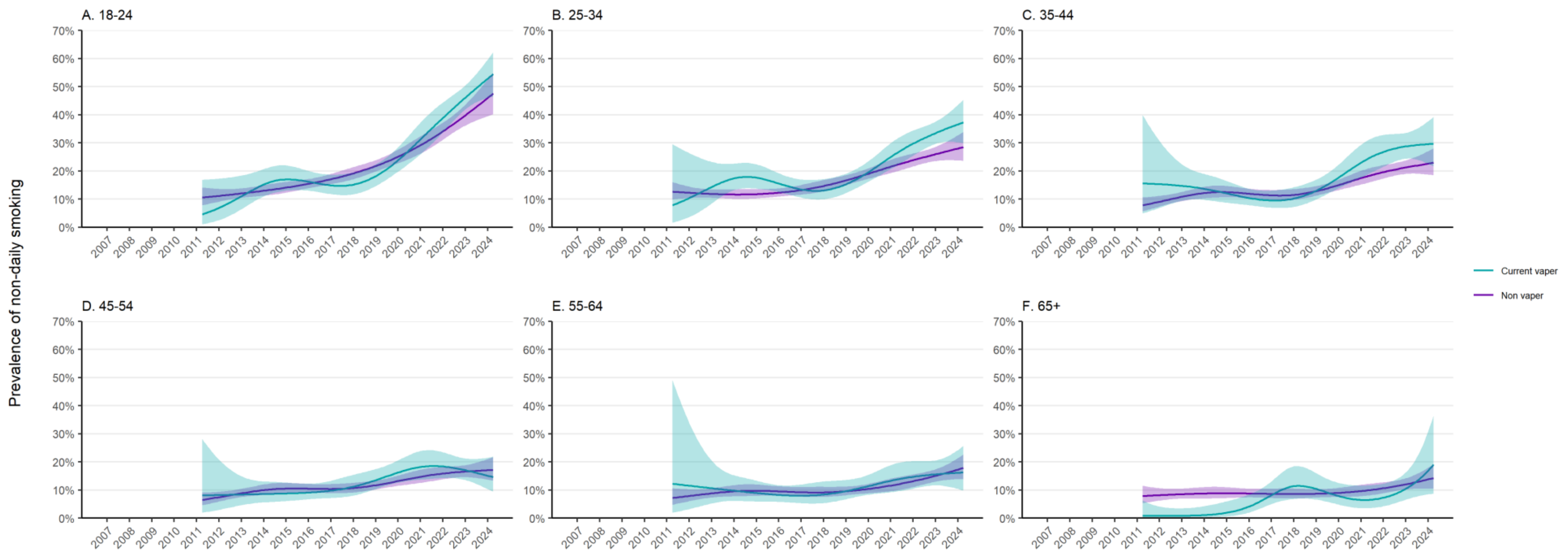

Figure S6. Trends by vaping status in non-daily smoking among adult cigarette smokers in England, stratified by age.

Lines represent the modelled weighted proportion by monthly survey wave (modelled non-linearly using restricted cubic splines; five knots) and vaping status, stratified by age group. Shaded bands represent 95% confidence intervals. Note: data on vaping status were only available for cigarette smokers from April 2011.

Unweighted sample sizes: 18-24 *n*=7,234; 25-34 *n*=8,882; 35-44 *n*=7,161; 45-54 *n*=7,153; 55-64 *n*=6,106; 65+ *n*=5,714.

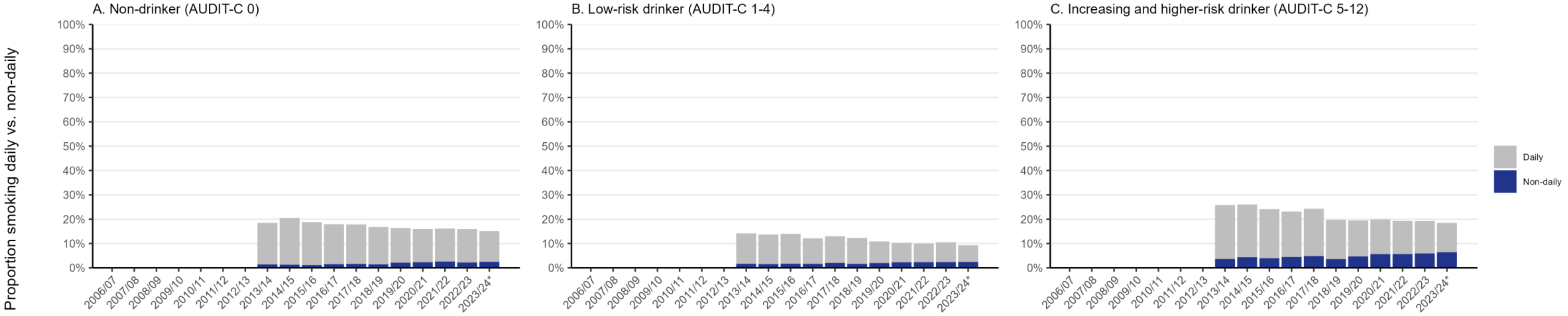

Figure S7. Prevalence of daily and non-daily smoking, by level of alcohol consumption.

Panels show weighted data aggregated by year among adults in England. Bars represent the proportions smoking daily and non-daily. Data are aggregated across 12-month periods (November to October). Data on alcohol consumption were collected from April 2014. *Data for 2023/24 are based on November to April only.

Table S3. Strength of urges to smoke among non-daily smokers in England, by vaping status

	Year[1]					
	**2006-09**	**2009-12**	**2012-15**	**2015-18**	**2018-21**	**2021-24**
**Non vapers (exclusive smokers)**						
Mean [95% CI]	-	1.2 [1.2–1.3]	1.1 [1.0–1.1]	0.9 [0.9–1.0]	0.9 [0.8–1.0]	0.9 [0.8–1.0]
0. Not at all	-	31.6 [28.8–34.5]	38.8 [35.5–42.2]	46.9 [43.8–50.0]	45.7 [42.6–48.9]	46.1 [42.8–49.5]
1. Slight	-	26.2 [23.6–28.9]	25.9 [23.0–29.1]	23.5 [21.0–26.2]	27.6 [24.8–30.5]	25.7 [22.9–28.7]
2. Moderate	-	31.6 [28.8–34.4]	26.3 [23.4–29.4]	22.9 [20.4–25.6]	20.3 [17.9–22.9]	21.7 [19.0–24.6]
3. Strong	-	8.0 [6.5–9.8]	6.9 [5.4–8.7]	4.4 [3.2–6.0]	4.2 [3.1–5.7]	3.8 [2.8–5.3]
4. Very strong	-	1.7 [1.1–2.7]	2.1 [1.3–3.3]	1.7 [1.1–2.7]	1.4 [0.9–2.4]	2.2 [1.4–3.5]
5. Extremely strong	-	0.9 [0.5–1.7]	0.1 [0.0–0.4]	0.6 [0.3–1.3]	0.7 [0.3–1.7]	0.4 [0.2–1.2]
**Current vapers (dual users)**						
Mean [95% CI]	-	2.1 [1.8–2.4]	1.5 [1.3–1.7]	1.7 [1.5–1.8]	1.4 [1.2–1.5]	1.4 [1.3–1.5]
0. Not at all	-	21.4 [16.3–27.5]	16.6 [12.4–22.0]	23.5 [18.8–28.8]	24.4 [20.9–28.3]	11.4 [3.1–33.7]
1. Slight	-	28.7 [22.9–35.3]	23.8 [18.6–29.9]	31.1 [25.9–36.9]	30.0 [26.1–34.2]	68.4 [43.7–85.8]
2. Moderate	-	34.9 [28.6–41.8]	40.8 [34.5–47.3]	36.6 [31.1–42.6]	30.9 [27.0–35.0]	17.7 [5.7–43.2]
3. Strong	-	9.9 [6.6–14.6]	13.9 [10.1–18.9]	5.5 [3.5–8.5]	11.8 [9.2–15.1]	2.6 [0.3–19.3]
4. Very strong	-	3.7 [1.7–8.2]	3.5 [1.8–7.0]	2.2 [0.9–5.1]	2.2 [1.3–3.7]	1.4 [0.4–4.8]
5. Extremely strong	-	1.3 [0.5–3.6]	1.2 [0.4–3.3]	0.7 [0.2–2.3]	1.2 [1.2–1.3]	1.1 [1.0–1.1]

Data are shown as column percentages with 95% CI, unless otherwise specified.
[1] Years are coded from November to October (i.e., November 2009 to October 2012, etc.). Note 2021-24 includes data from November 2021 to April 2024 only. No data are provided for 2006-2009 because vaping status was not assessed before April 2011.
